# Supplementary material for: Multi-omic profiling of pathogen-stimulated primary immune cells
Source: iScience. 2024 Jul 6;27(8):110471. doi: 10.1016/j.isci.2024.110471 (PMC11293528; doi:10.1016/j.isci.2024.110471)
Supplement: Document S1. Figures S1–S12 and Table S17 [file mmc1.pdf]

iScience, Volume *27*

Supplemental information

Multi-omic profiling of pathogen-stimulated primary immune cells

Renee Salz, Emil E. Vorsteveld, Caspar I. van der Made, Simone Kersten, Merel Stemerdink, Tabea V. Riepe, Tsung-han Hsieh, Musa Mhlanga, Mihai G. Netea, Pieter-Jan Volders, Alexander Hoischen, and Peter A.C. 't Hoen

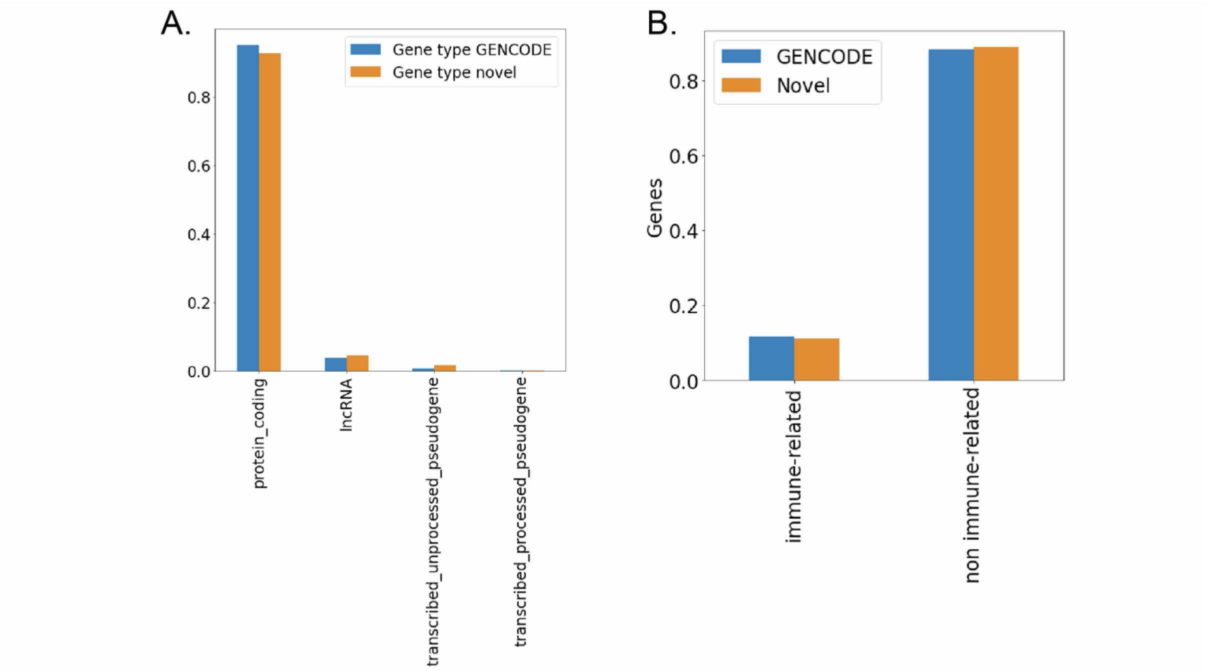

Figure S1. Gene types where novel transcripts are found, related to Figure 2. A) Most common categories of genes. B) Categorization by genes that are related to immunity. See also Table S26.

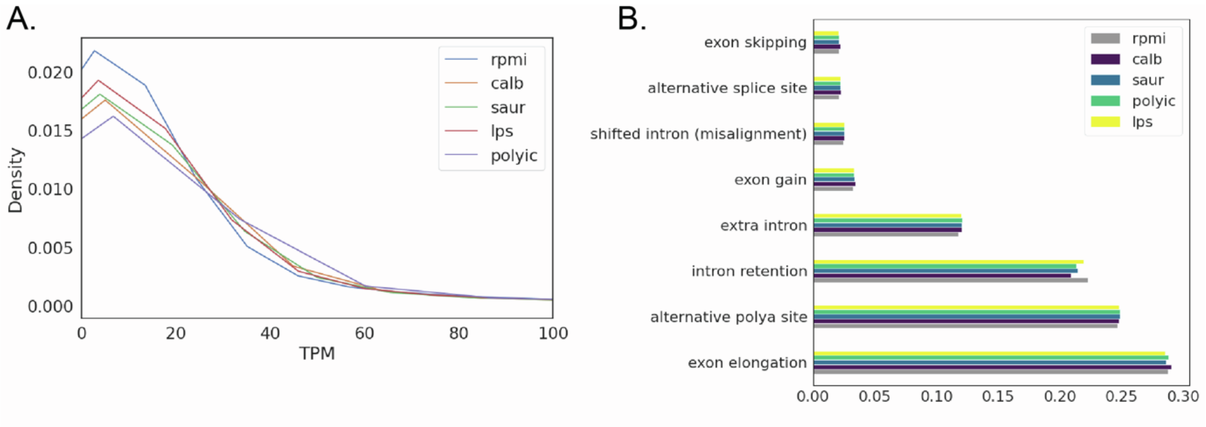

Figure S2. Transcriptome trends in all conditions, related to Figure 2. A) TPM distribution for each of the conditions measured. B) Ratios of novelty events in the transcriptome by condition. See also Table S26.

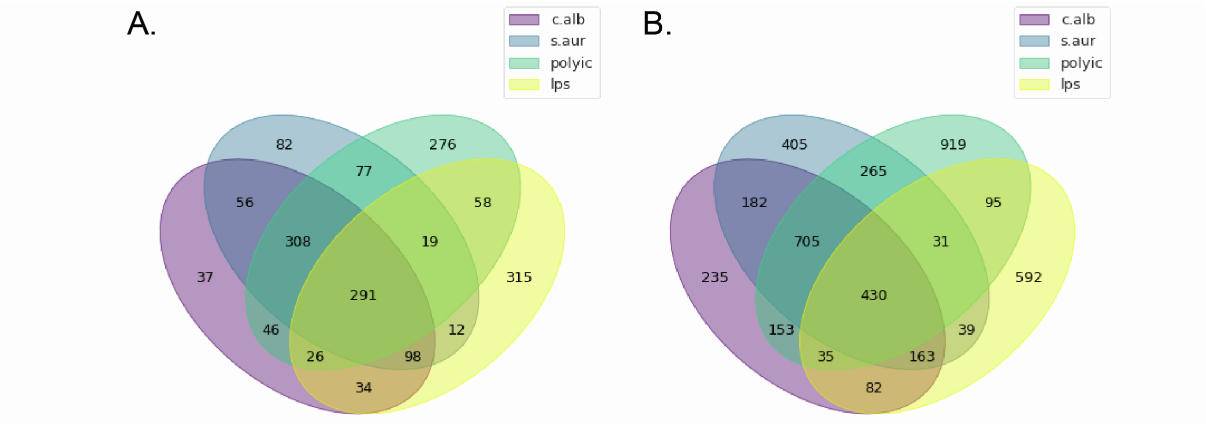

Figure S3. Differential expression on the RNA level, related to Figure 3. A) Differentially expressed genes. B) Differentially expressed transcripts. See also Tables S1 and S2.

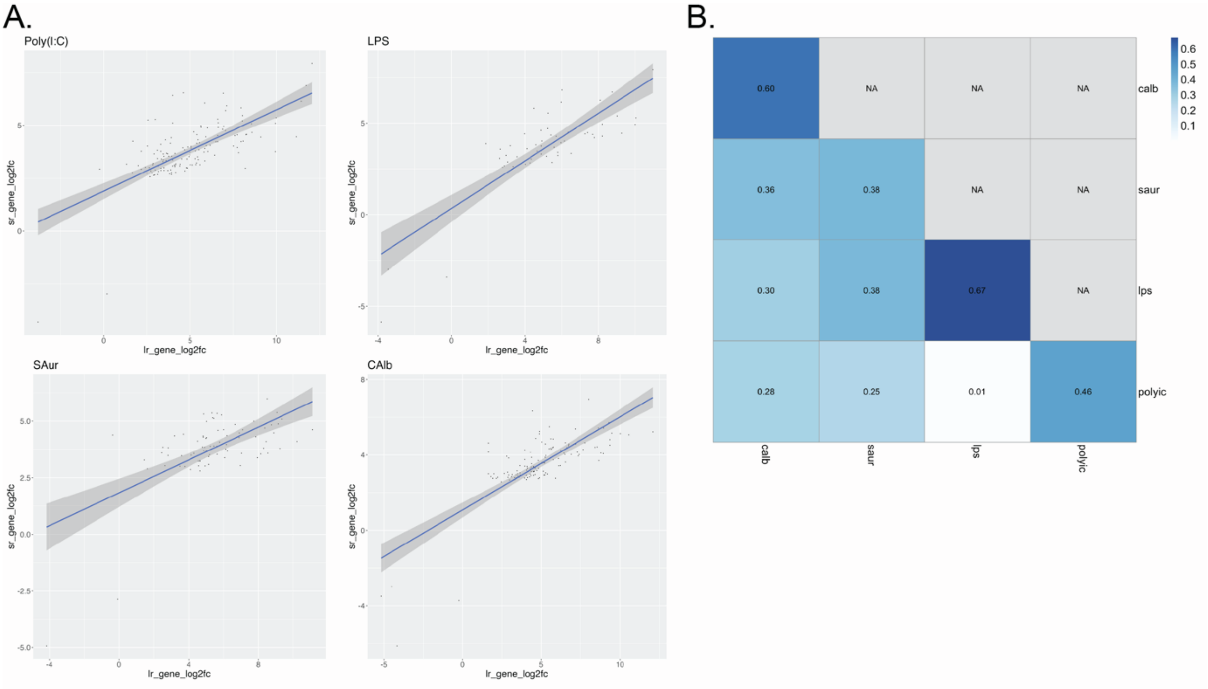

Figure S4. Differential gene expression validation using short read sequencing (QuantSeq), related to STAR methods. A) Correlation of DEGs of each condition between long- and short-read sequencing. B) Correlation between long-and short-read sequencing when comparing each condition to each other. See also Table S3.

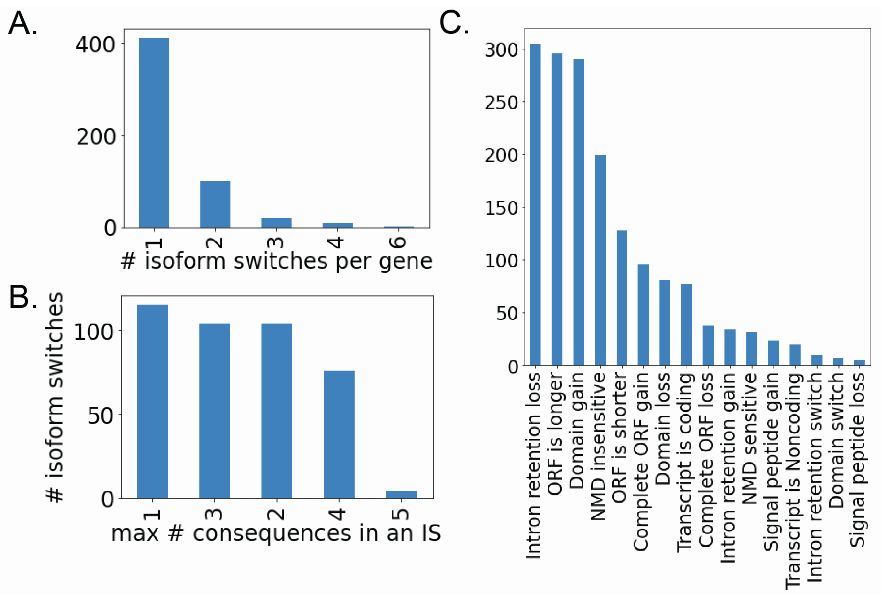

Figure S5. Overview of isoform switching, related to Figure 4. D) Number of isoform switches occurring per gene. E) Max number of measured consequences per isoform switch. F) Total number of each type of isoform switching consequence. See also Tables S5 and S6.

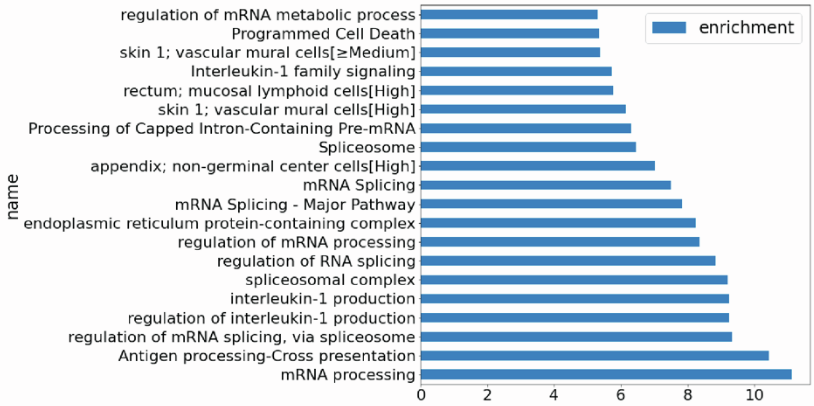

Figure S6. Pathway analysis of genes affected by intron retention loss, related to Figure 4. Enrichment was calculated using gProfiler using 304 intron retention loss isoform switches from 145 genes. See also Tables S12 and S13.

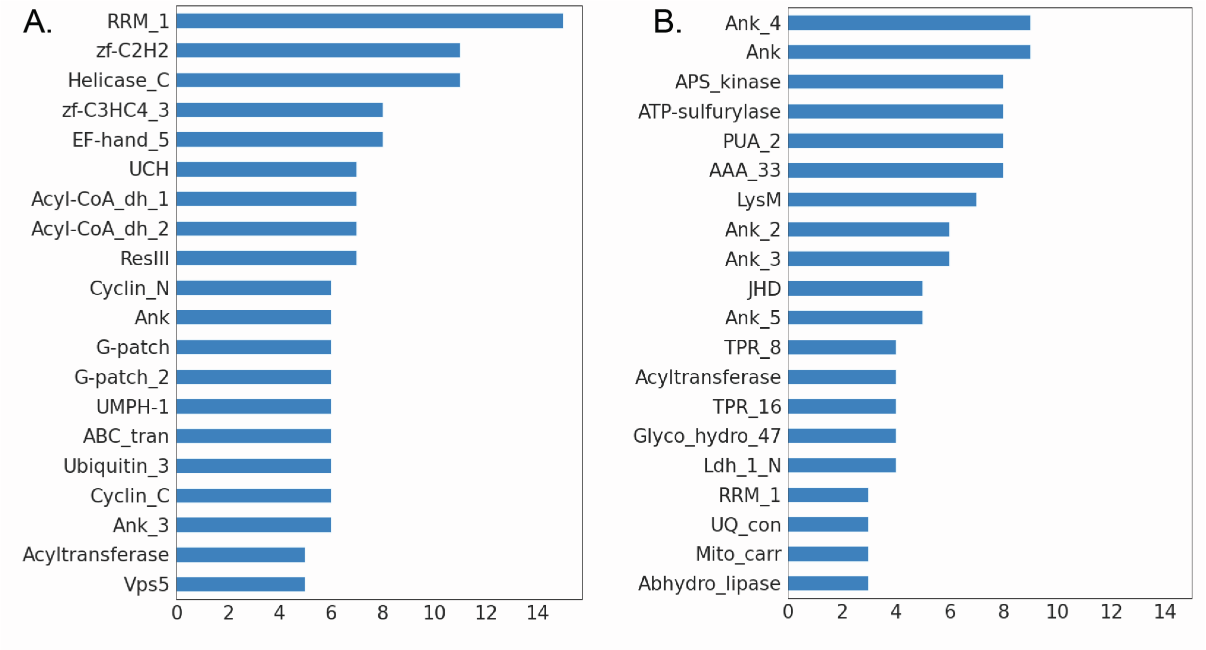

Figure S7. Domains that were most frequently gained/lost, related to Figure 4. A) Domains gained. B) Domains lost. See also Tables S14-S16.

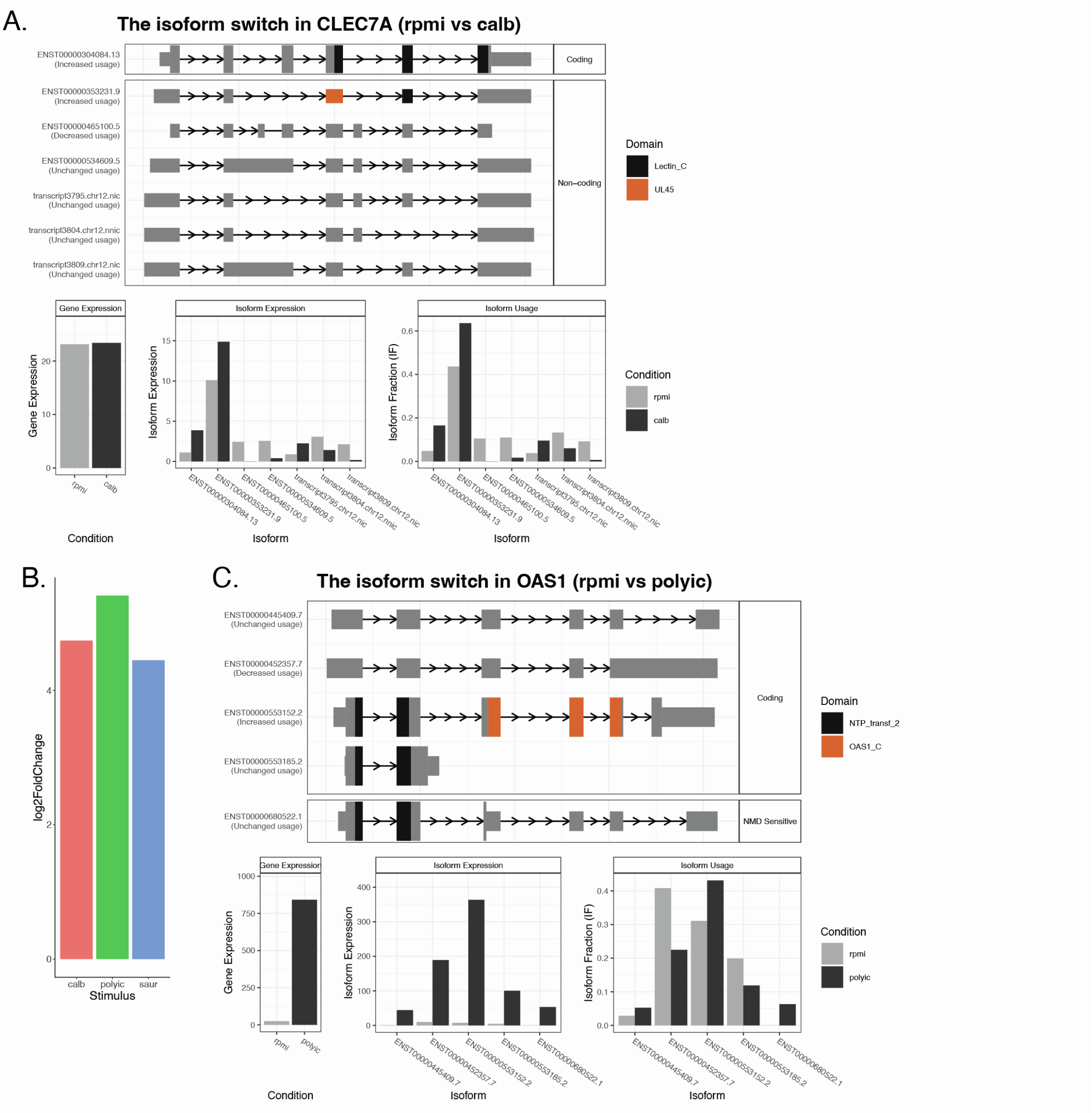

Figure S8. Examples of stimulus-specific isoform switching, related to Figure 4. A) isoform switching in CLEC7A specific to C. albicans stimulation. B) Differential expression of OAS1 for three stimuli. C) isoform switching of OAS1 specific to Poly(I:C) stimulation. See also Tables S5 and S6.

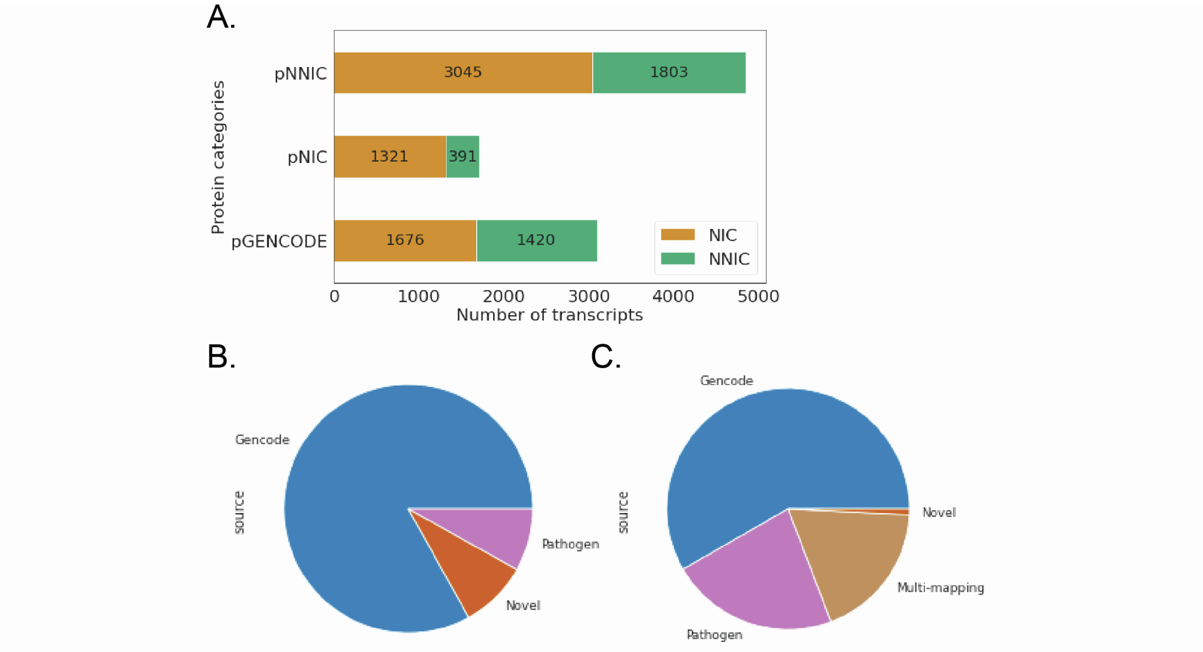

Figure S9. Open reading frames resulting from novel transcripts and their detectability, related to Figure 7. A) RNA vs predicted protein novelty. Color indicates RNA novelty category. X-axis corresponds to unique transcripts. B) (Predicted) protein sequences that comprise the full search database. C) Unique peptide sequences in the peptide search database by origin. Multi-mapping indicates 2 or more possible origin categories. See also Table S27 and STAR methods.

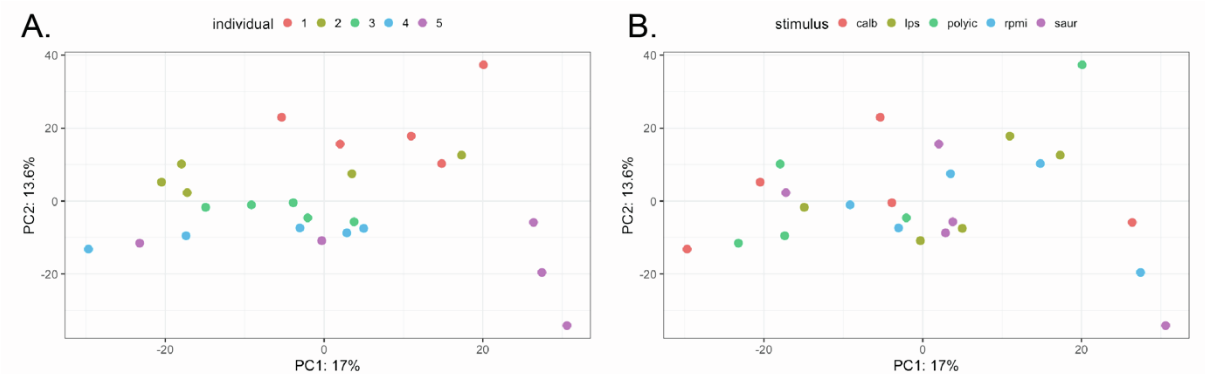

Figure S10. Principal component analysis of peptide identifications on the secretome of all samples, related to Figure 7. A) Colored by individuals 1-5. B) Colored by stimulus condition. See also Tables S26 and S27.

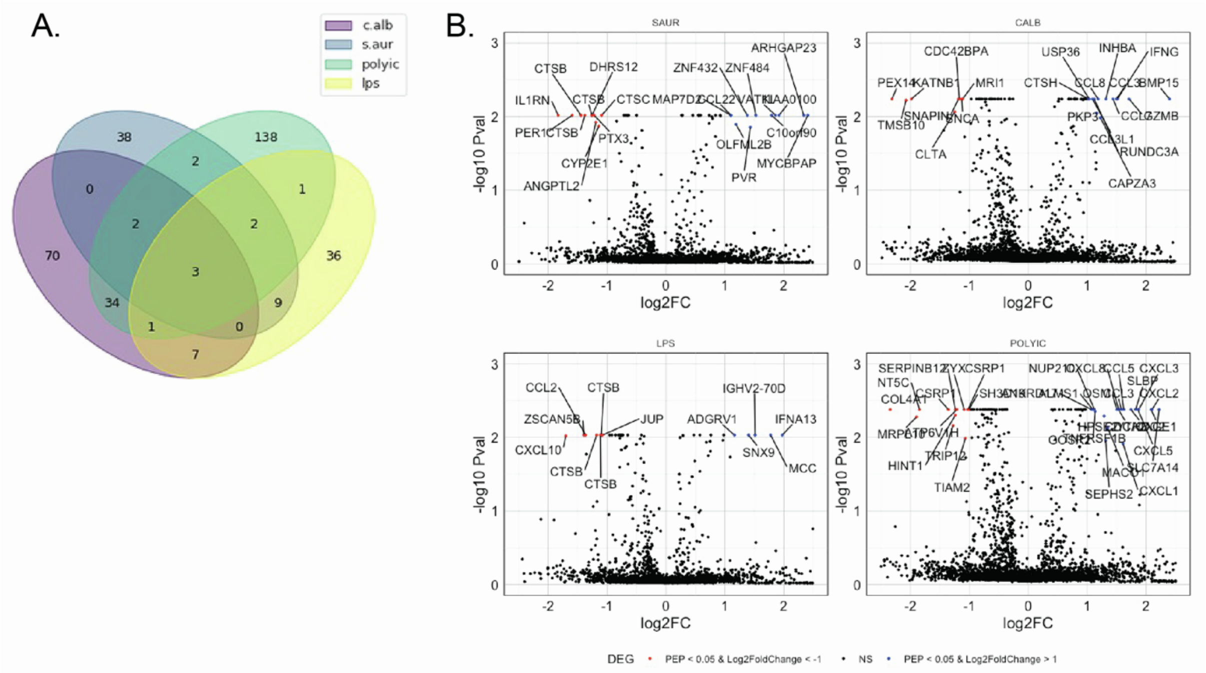

Figure S11. Differential expression on the secretome, related to Figure 7. A) Differentially expressed proteins by condition. B) Differentially expressed protein volcano plots per condition. See also Tables S26 and 27.

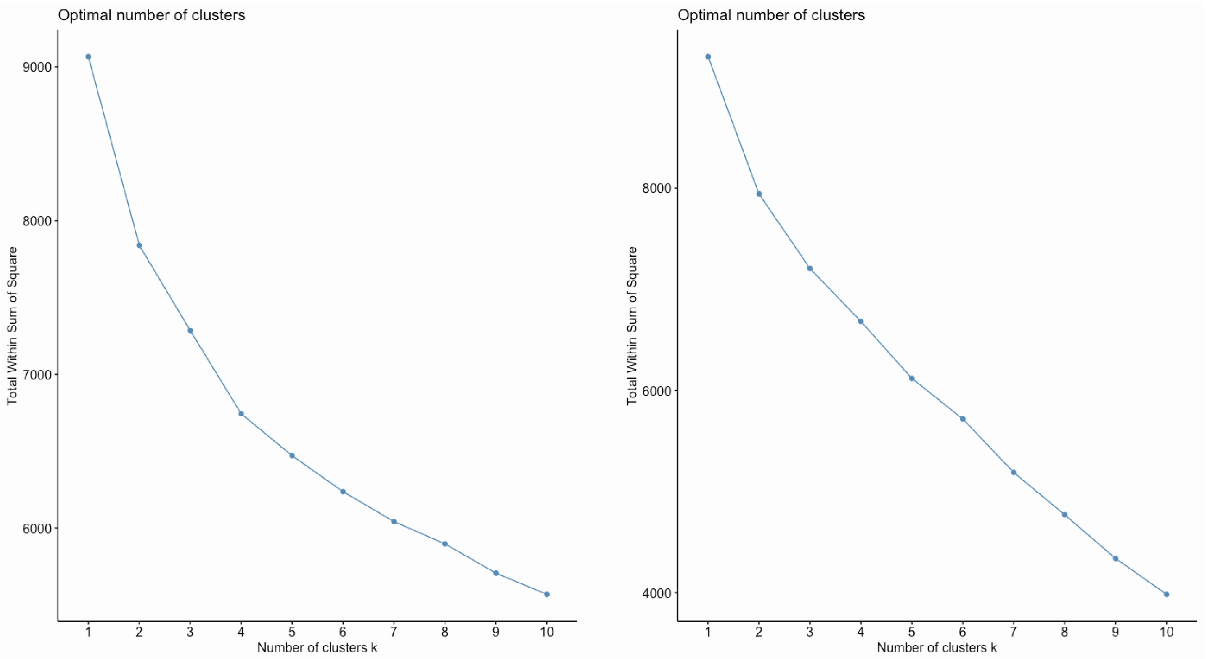

Figure S12. Elbow plots using kmeans clustering, related to Figure 7. Elbow plots to determine the optimal number of kmeans clusters to use for clustering of proteomics data. See also Tables S26 and S27.

Table S17. Transcript novelty combination counts per isoform switch consequence, related to Figure 4.

switchConsequence	both known	both novel	novel down	novel up
Complete ORF gain	93	4	47	8
Complete ORF loss	21	1	14	14
Domain gain	178	26	206	12
Domain loss	45	8	21	35
Domain switch	1	0	5	3
Intron retention gain	9	3	24	9
Intron retention loss	138	24	269	20
Intron retention switch	0	0	11	1
NMD insensitive	51	17	227	1
NMD sensitive	4	6	1	30
ORF is longer	149	28	265	2
ORF is shorter	80	12	20	41
Signal peptide gain	21	1	10	3
Signal peptide loss	4	0	0	6
Transcript is Noncoding	13	0	6	7
Transcript is coding	67	1	43	7
